# Reply to Bourgeois, P. Comment on “Devoogdt et al. The Effectiveness of Fluoroscopy-Guided Manual Lymph Drainage as Part of Decongestive Lymphatic Therapy on the Superficial Lymphatic Architecture in Patients with Breast Cancer-Related Lymphoedema: A Randomised Controlled Trial. *Cancers* 2023, *15*, 1545”

**DOI:** 10.3390/cancers16132435

**Published:** 2024-07-02

**Authors:** Wiebren A. A. Tjalma, Jean-Paul Belgrado, Sarah Thomis, Ines Nevelsteen, Nick Gebruers, Chris Monten, Marianne Hanssens, Tessa De Vrieze, Nele Devoogdt

**Affiliations:** 1 Multidisciplinary Breast Clinic and Multidisciplinary Oedema Clinic, Antwerp University Hospital, 2650 Antwerp, Belgium; 2 Faculty of Medicine and Health Sciences, University of Antwerp, MIPRO, 2610 Antwerp, Belgium; 3 Lymphology Research Unit, Université Libre de Bruxelles, 1070 Brussels, Belgium; 4 Department of Vascular Surgery, Centre for Lymphedema, UZ Leuven—University Hospitals Leuven, 3000 Leuven, Belgium; 5 Department of Cardiovascular Sciences, KU Leuven—University of Leuven, 3000 Leuven, Belgium; 6 Multidisciplinary Breast Centre, UZ Leuven—University Hospitals Leuven, 3000 Leuven, Belgium; 7 Department of Rehabilitation Sciences and Physiotherapy, University of Antwerp, MOVANT, 2610 Antwerp, Belgium; 8 Department of Radiotherapy, Ghent University Hospital, 9000 Ghent, Belgium; 9 Department of Oncology, Centre for Oncology, General Hospital Groeninge, 8500 Kortrijk, Belgium; 10 Department of Rehabilitation Sciences, KU Leuven—University of Leuven, 3000 Leuven, Belgium

We appreciate the commentary by Pierre Bourgeois [1] regarding our published article [2]. Breast cancer-related lymphedema (BCRL) is one of the most feared complications due to its chronicity [3]. The pooled incidence of BCRL in the upper limb is 16.6%, with a range of 11.8–53.5% for axillary lymph node dissection (ALND) and 0–15.8% for sentinel lymph node (SLN) procedures [4,5,6]. The risk is twice as high in black women as in white women [7]. Despite the decreasing incidence, the prevalence of BCRL is increasing, attributed to improved survival rates. Previously, lymphoscintigraphy was the reference standard imaging technique for lymphedema; however, indocyanine green (ICG) lymphofluoroscopy is now considered the most informative lymphatic imaging technique [8,9,10].

We thank Dr. Bourgeois for his clear interest in our research on the role of manual lymph drainage (MLD) in improving the superficial lymphatic architecture in patients with chronic mild-to-moderate BCRL [2,11]. Especially his concern about ICG lymphofluoroscopy is remarkable, with this letter being the third in a row on the same topic [11,12,13,14,15,16].

Dr. Bourgeois raises the question whether ICG may have a toxic effect on the lymphatic system, which could potentially explain the results of our study. He based his concern on the observation that the superficial lymphatic architecture did not differ among the three treatment groups (i.e., “placebo” versus traditional MLD versus fluoroscopy-guided MLD), despite a decrease in the dermal backflow score and a decrease in the number of lymph nodes for each of the groups during follow-up.

ICG was indeed used to visualize the superficial lymphatic architecture. In healthy subjects, ICG lymphofluoroscopy reveals a linear lymph transport pattern. However, in patients with BCRL, three dysfunctional backflow patterns (splash, stardust and diffuse) of lymphatic transport can be discerned [10]. This retrograde lymph transport leads to a dermal rerouting pattern on ICG lymphofluoroscopy. The dermal rerouting score calculation is based on the severity and extensiveness of the retrograde lymph transport. A decrease in this score indicates an improvement in lymph transport, as observed in our trial. Therefore, we find it perplexing how colleague Bourgeois interprets this as a deterioration of the lymph transport. A significant decrease in the number of lymph nodes occurred during maintenance treatment (not significantly different compared to baseline). Although there was a statistically “significant” result during intensive treatment, it cannot be interpreted as a clinically relevant change.

The presence or absence of a lymph node should not be viewed as motivation for any potential toxic behaviour of ICG. If lymph transport is directed towards the deeper lymphatic system, no node is visualized. Additionally, concerning the “toxic effect of ICG”, if this product decreases the lymphatic function, one would anticipate further impairment with additional injections. Again, this is not what we observed.

In studies investigating the potential toxic effects of ICG on the lymphatic function, Gashev et al. and Weiler et al. indeed concluded that there was a decreased lymphatic function in ex vivo mesenteric lymphatic rat vessels and in in vivo rat tail lymphatic vessels [17,18]. However, Weiler et al. also noted that the very low concentrations of ICG made it challenging to draw any relevant conclusions about the impact on the lymphatic vessels [18]. The opposite was reported by Aldrich et al., where they injected ICG at various concentrations into mice tails and found no significant change in lymphatic propulsive velocity or frequency [19]. Although these findings are useful, one must keep in mind that lymphatic vessels in humans differ from rodents. Consequently, as in all research, findings from animal experiments cannot simply be interpreted as clinically relevant in a human population.

Moreover, ICG has been used in humans for several decades and is considered a safe and effective way to identify small lymphatic vessels [9,20]. ICG has become the gold standard for the identification of lymph vessels during microscopic surgery for lymphovenous anastomosis in the treatment of secondary lymphedema [21]. Although lymphoscintigraphy may also be used to identify lymphatic vessels during such procedures, the radiocolloids come with some considerable disadvantages. Firstly, it is an ionizing product, inherently exposing the patient and health-care provider to radiation, however small this may be. Secondly, the poor spatial and temporal resolution of the radiocolloids limits its ability to visualize the smaller lymphatic vessels and cannot visualize lymphatic propulsion activity within conducting vessels [22].

In conclusion, the potential toxicity of ICG to the lymphatic vessels in rodents has not been reported in humans. Furthermore, ICG lymphofluoroscopy offers superior visualization of the lymphatic architecture compared to radiocolloids, particularly for smaller lymph vessels, and poses a lower risk to both patients and caregivers. It is worth noting that our study maintained strict methodology, especially considering anticipated resistance to findings that challenge long-standing habits and assumptions.

We greatly appreciate the concerns raised by our colleague Bourgeois regarding ICG lymphofluoroscopy and are pleased to address them fully. Changing habits in medicine requires not only compelling evidence of efficacy but also effective communication, education, and support to encourage the adoption of new practices. Our randomized controlled trial demonstrated that MLD offers no added value for patients with chronic to moderate BCRL. RCTs are assigned the highest level of evidence. We hope that this letter contributes to the integration of this paradigm shift into everyday practice.
